# Multifocal bilateral desmoid tumour of perirenal tissues with peribiliary localization

**DOI:** 10.1259/bjrcr.20150099

**Published:** 2015-12-16

**Authors:** Beatrice Sacconi, Renato Argirò, Angelo Iannarelli, Alessandro Di Gaeta, Mario Bezzi

**Affiliations:** Department of Radiological, Oncological and Anatomopathological Sciences, Sapienza University of Rome, Rome, Italy

## Abstract

Desmoid tumour (DT) is an unusual, benign tumour, more frequently observed in patients with familial polyposis and pregnant females. It usually presents as a single mass lesion, more frequently showing a compressive rather than an infiltrative growth pattern. We report a case of a 70-year-old male presenting with a multifocal, bilateral infiltrative DT of the perirenal tissue, with involvement of the choledochus wall. The patient was partly treated with tamoxifen and docetaxel, but both therapies were discontinued in accordance with the patient’s decision owing to mild toxicity; however, a CT examination performed 3 months later showed an unexpected remarkable reduction of the tumour at all sites. At 1 year follow-up, new pathologic tissue was visible surrounding the right renal pelvis and the calices.

## Summary

Desmoid tumour (DT) is an unusual, benign tumour, more frequently observed in patients with familial polyposis and pregnant females. It usually presents as a single mass lesion, more frequently showing a compressive rather than an infiltrative growth pattern. We report a case of a 70-year-old male presenting with a multifocal, bilateral infiltrative DT of the perirenal tissue, with involvement of the choledochus wall. The patient was partly treated with tamoxifen and docetaxel, but both therapies were discontinued in accordance with the patient’s decision owing to mild toxicity; however, a CT examination performed 3 months later showed an unexpected remarkable reduction of the tumour at all sites. At 1 year follow-up, new pathologic tissue was visible surrounding the right renal pelvis and the calices.

## Case report

**Figure 1.**
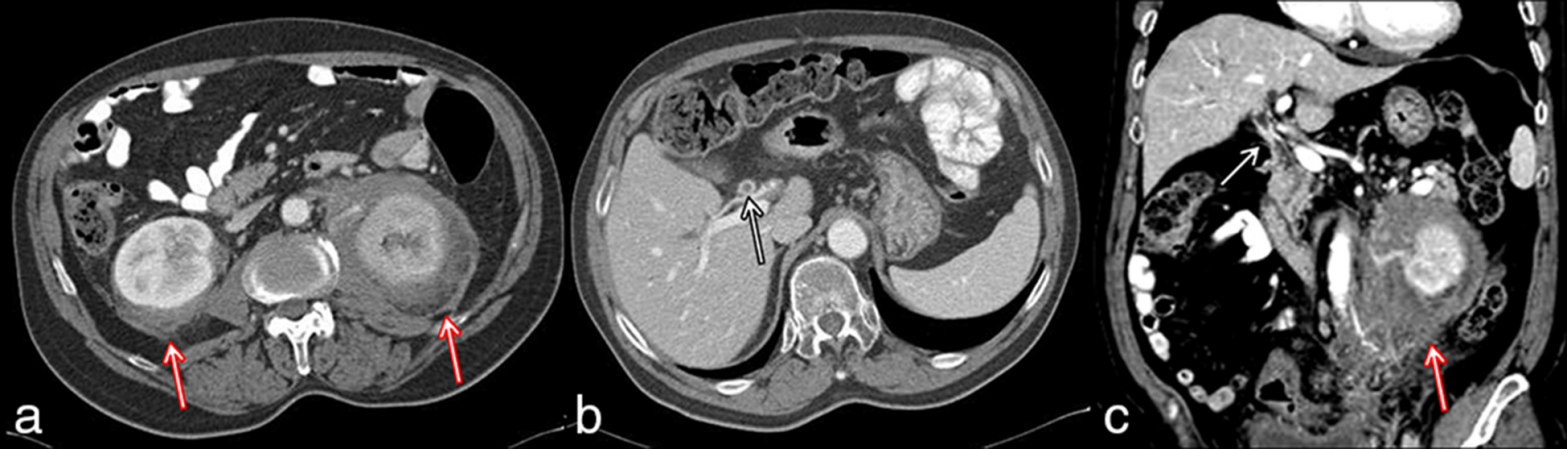
Axial portal-phase CT scan showing (a) a large amount of solid tissue in the left perirenal space and a smaller amount on the contralateral side (red arrows) and (b) a partial stenosis of the common bile duct owing to hypervascular eccentric tissue (white arrow). (c) Coronal portal-phase CT image showing left perirenal (red arrow) and peribiliary localization (white arrow).

**Figure 2.**
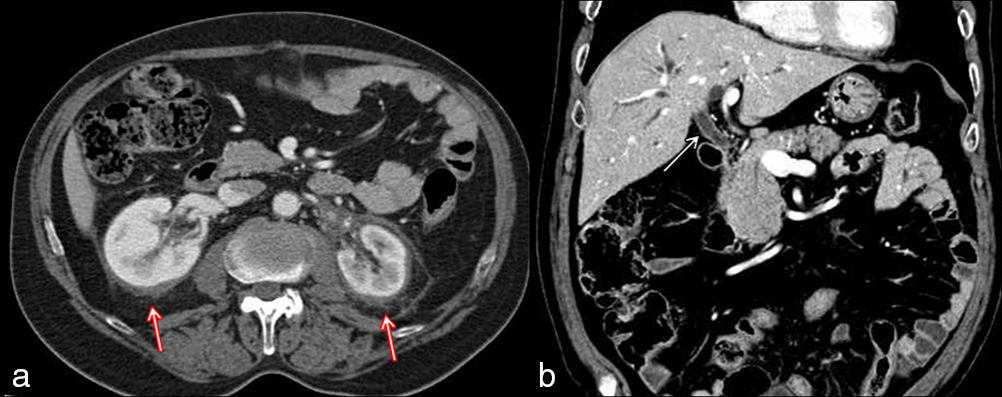
(a) Axial portal-phase CT scan performed after 3 months of medical therapy shows a marked reduction in size of the perirenal tissue on both sides (red arrows). (b) Coronal portal-phase CT image showing a similar reduction in size of the peribiliary tissue (white arrow); a residual ectasia of intrahepatic bile ducts was observed.

**Figure 3.**
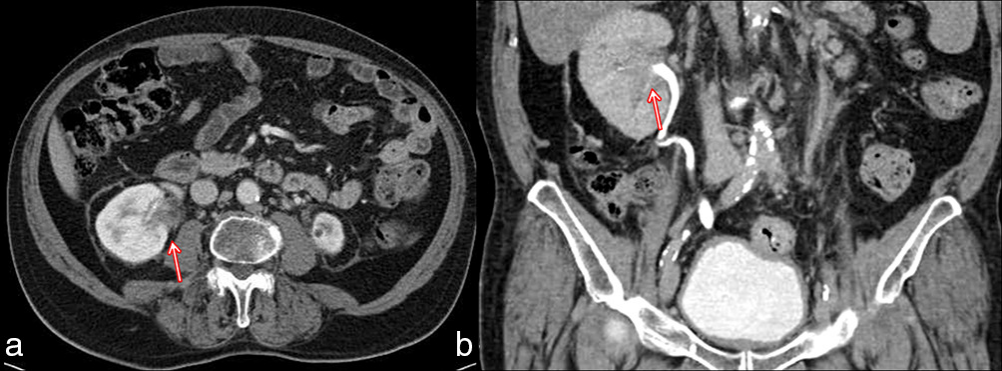
(a) Axial portal-phase CT scan performed 1 year later showing new solid tissue surrounding the right renal pelvis and calices (red arrow). (b) Coronal delayed-phase CT image confirming the presence of solid tissue infiltrating the renal pelvis (red arrow).

A 70-year-old male was referred to our hospital complaining of left flank pain. At physical examination, nothing relevant was observed and laboratory findings were within normal limits. A CT examination of the abdomen and the pelvis was performed with a multidetector scanner, before and after contrast media administration. Portal phase images showed a large amount of solid tissue in the left perirenal space, infiltrating the renal capsule and the main renal vessels; the tissue did not show significant contrast enhancement. Similar findings were detected also in the right perirenal space (Figure 1a,c). CT images also revealed a partial stenosis of the common bile duct, with intrahepatic bile duct ectasia owing to hypervascular eccentric tissue (Figure 1b,c). Hence, a diagnostic integration with endoscopic retrograde cholangiopancreatography was performed to exclude an intraductal proliferation. A biopsy was also performed in the left perirenal space; the pathological samples were composed of connective and adipose tissues, revealing the histological features of a DT or abdominal fibromatosis and also showing immunohistochemical markers typical of muscular tissues, such as actine. The tumour was considered unresectable and medical therapy was started with tamoxifen (20 mg die^–1^); after an episode of thrombophlebitis, the patient asked to suspend tamoxifen and accepted a new therapeutic regimen (docetaxel 75 mg m^−2^ every 3 weeks); unfortunately, even this treatment was discontinued after only 4 weeks owing to neuropathy. A new CT examination was then performed to assess the results of the treatment. Unexpectedly, on venous phase images, the perirenal tissue showed a remarkable reduction on the left side and had almost disappeared on the right side (Figure 2a). The peribiliary tissue had equally decreased in size and thickness (Figure 2b). The treatment was then discontinued in accordance with the patient’s decision. 1 year later, both the left perirenal and peribiliary tissues demonstrated no progression and remained clinically stable on off-treatment; however, new tissue was visible surrounding the right renal pelvis and the calices (Figure 3a,b). The same therapeutic regimens (tamoxifen and docetaxel) were proposed to the patient based on the previous good response; unfortunately, the patient refused any treatment.

## Discussion

DT is a benign neoplasm, derived from a monoclonal proliferation of myofibroblasts.^1–4^ Although its pathogenesis is still unclear, DT has a higher incidence in patients with familial polyposis or Gardner syndrome (10–15%), associated with specific mutations of the *APC* gene.^5^ The general incidence of DT in patients without a history of familial polyposis, such as in our patient, is 2–4 cases per million, with a sex ratio from 2 : 1 to 5 : 1. Sporadic DTs are more commonly observed in pregnant females (owing to a much-discussed role of oestrogens in pathogenesis) and in patients with previous major surgery.^1–4^ Our patient had previously undergone an exploratory laparotomy for gastric pain, cholecystectomy and hernioplasty 35, 30 and 15 years ago, respectively. DT usually does not metastasize and has a variable prognosis, alternating periods of progression and remission, and also shows a high tendency of recurrence after local excision. According to their localization, DTs can be classified as extra-abdominal (frequently localized in the thoracic wall and the shoulders, and confined to the muscles and the aponeurosis) and abdominal. Abdominal tumours can have a superficial or intra-abdominal localization.^1–4^ The DT described in this report showed an unusual bilateral (both perirenal spaces) as well as multifocal localization (peribiliary space); furthermore, the tumour had an infiltrative growth pattern, whereas most DTs compress contiguous structures, without real infiltration.^3^

Imaging techniques are mainly used to define the tumour’s dimensions and the infiltration of contiguous structures to plan surgery. These tumours usually show considerable heterogeneity at imaging; as a matter of fact, different lesions, even in the same patient, rarely showed identical appearance, depending on the relative amounts of fibroblast proliferation, fibrosis, collagen content and vascularity. On ultrasound, DTs demonstrate low, medium or high echogenicity, with ill-defined borders.^6^ On CT scan, they can be either ill-defined or well circumscribed, with variable attenuation relative to muscle, and may or may not show enhancement after contrast media administration.^6,7^ MRI demonstrates low signal intensity relative to muscle on *T*_1_ weighted images, variable signal intensity on *T*_2_ weighted images and variable contrast enhancement. Low *T*_2_ signal intensity bands can represent foci of high concentrations of collagen deposition.^6^ In our case, the perirenal tissue was hypodense and ill-defined and did not show significant contrast enhancement; on the contrary, the tissue around the wall of the choledochus was markedly hyperdense after contrast injection.

Owing to this variable appearance, making a definitive diagnosis requires histological examination of the tissue samples. DTs are usually encapsulated unmoving lesions, similar to cicatricial tissues; an infiltrative growth pattern is less frequent. Microscopically, they consist of small, oblong cells with pale nucleus without atypias, separated by abundant collagen; macrophages, giant cells and lymphocytes are observed in peripheral areas. Moreover, DTs show expression of immunohistochemical markers typical of muscular tissues, such as actine, desmine and vimentin.^3^ Clinical signs and symptoms depend on the tumoral site, dimensions and growth rate. DTs usually present as palpable or visible masses; pain and paraesthesia can be caused by compression of the nerves or muscles. In case of abdominal localization, severe complications, such as intestinal or ureteral obstruction, and fistulization, can occur, with worse prognosis.^1–3^

There is no appropriate treatment for any DT; most authors currently advocate for an individualized/multimodality approach.^8^ Clinical and radiological monitoring with CT scan can be used for small and slowly-progressing tumours. Otherwise, surgery is the main treatment modality; the resection has to be wide, and the margin status seems to be the most important predictor of local recurrence.^8,9^ If the tumour infiltrates vital organs or structures, the main treatment modality is medical therapy consisting of administration of anti-inflammatories (such as celecoxib and sulindac) and the modulation of oestrogenic receptors owing to the recently admitted role of oestrogens in the pathogenesis.^10^ Approximately, 50% of DTs show complete or partial response to medical approach; in the presented case, we can presume that the tumour responded to tamoxifen, as such a good response would not have been likely after 4 weeks of docetaxel. Medical therapy can be used as an adjuvant to surgery; several trials have demonstrated that a combined strategy could reduce local recurrence, especially in advanced intra-abdominal disease. Chemotherapy and radiotherapy are also used in selected cases of unresectable tumours or as an adjuvant to surgery; in this regard, several tyrosine kinase inhibitors (such as imatinib or sorafenib) and cytotoxic chemotherapy (doxorubicin, dacarbazine, docetaxel, vinorelbine, vinblastine and gemcitabine) have been associated with clinical benefit in patients affected by DTs; however, this topic is still being debated, especially regarding the best chemotherapy regimens to be used. As a matter of fact, in the literature, reported results are mainly based on small single-centre case series studies; furthermore, in all these studies, the authors could not really affirm that disease stability was related to a benefit from chemotherapy and not simply to the natural history of the tumour.^2,3,6–8^ In our case, the patient’s poor compliance considerably influenced the medical treatment, which was intermittently and incompletely performed; nevertheless, this unusual bilateral multifocal tumour surprisingly showed a very remarkable response to therapy.

## Learning points

1. DTs are benign neoplasms caused by monoclonal proliferation of myofibroblasts, more frequently occurring in patients with familial polyposis or Gardner syndrome, pregnant females and patients with previous major surgery.
2. DTs can be classified as extra-abdominal and abdominal. Abdominal tumours can have a superficial or intra-abdominal localization, usually presenting as a single mass lesion.
3. DTs usually compress contiguous structures, whereas infiltrative pattern of growth is less commonly observed.
4. DTs can show a variable appearance on imaging, depending on the relative amounts of fibroblast proliferation, fibrosis, collagen content and vascularity.
5. Surgery is the main treatment modality for DTs, even though most authors currently advocate for an individualized/multimodality approach.
6. DTs show a variable prognosis, alternating periods of progression and remission, with a high tendency of recurrence after local excision.
7. Medical therapy is mainly based on the administration of anti-inflammatories and the modulation of oestrogenic receptors, owing to the recognized role of oestrogens in the tumour pathogenesis.

## References

[1] Shinagare AB, Ramaiya NH, Jagannathan JP, Krajewski KM, Giardino AA, Butrynski JE, et al A to Z of desmoid tumors. AJR Am J Roentgenol 2011; 197: W1008–14. 22109314 10.2214/AJR.11.6657

[2] Berri RN, Baumann DP, Madewell JE, Lazar A, Pollock RE Desmoid tumor: current multidisciplinary approaches. Ann Plast Surg 2011; 67: 551–64. 21587055 10.1097/SAP.0b013e3182084cf6

[3] Kasper B, Ströbel P, Hohenberger P Desmoid tumors: clinical features and treatment options for advanced disease. Oncologist 2011; 16: 682–93. 21478276 10.1634/theoncologist.2010-0281 PMC3228186

[4] Sakorafas GH, Nissotakis C, Peros G Abdominal desmoid tumors. Surg Oncol 2007; 16: 131–42. 17719772 10.1016/j.suronc.2007.07.009

[5] Lips DJ, Barker N, Clevers H, Hennipman A The role of APC and beta-catenin in the aetiology of aggressive fibromatosis (desmoid tumors). Eur J Surg Oncol 2009; 35: 3–10. 18722078 10.1016/j.ejso.2008.07.003

[6] Casillas J, Sais GJ, Greve JL, Iparraguirre MC, Morillo G Imaging of intra- and extraabdominal desmoid tumors. Radiographics 1991; 11: 959–68. 1749859 10.1148/radiographics.11.6.1749859

[7] Einstein DM, Tagliabue JR, Desai RK Abdominal desmoids: CT findings in 25 patients. AJR Am J Roentgenol 1991; 157: 275–9. 1853806 10.2214/ajr.157.2.1853806

[8] de Bree E, Keus R, Melissas J, Tsiftsis D, van Coevorden F Desmoid tumors: need for an individualized approach. Expert Rev Anticancer Ther 2009; 9: 525–35. 19374605 10.1586/era.09.9

[9] Stoeckle E, Coindre JM, Longy M, Binh MB, Kantor G, Kind M, et al A critical analysis of treatment strategies in desmoid tumours: a review of a series of 106 cases. Eur J Surg Oncol 2009; 35: 129–34. 18760561 10.1016/j.ejso.2008.06.1495

[10] Hansmann A, Adolph C, Vogel T, Unger A, Moeslein G High-dose tamoxifen and sulindac as first-line treatment for desmoid tumors. Cancer 2004; 100: 612–20. 14745880 10.1002/cncr.11937

